# Supplementary material for: Toxic Effects of Perilla frutescens (L.) Britt. Essential Oil and Its Main Component on Culex pipiens pallens (Diptera: Culicidae)
Source: Plants (Basel). 2023 Mar 31;12(7):1516. doi: 10.3390/plants12071516 (PMC10096719; doi:10.3390/plants12071516)
Supplement: Supplementary file 1 [file plants-12-01516-s001.zip › plants-2253180-supplementary.pdf]

Supplementary materials

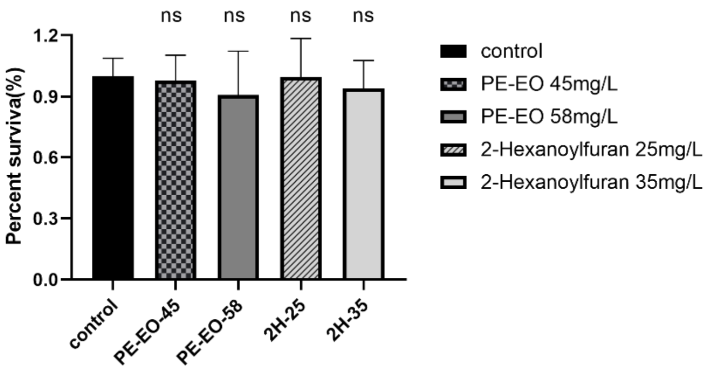

Figure S1. Effects of Perilla essential oil (PE-EO) and 2-hexanoylfuran against 297T cells. The data are given as Mean±SD. Abbreviation: ns, not signifcant (P>0.05) compared with the control according to Student's t-test.

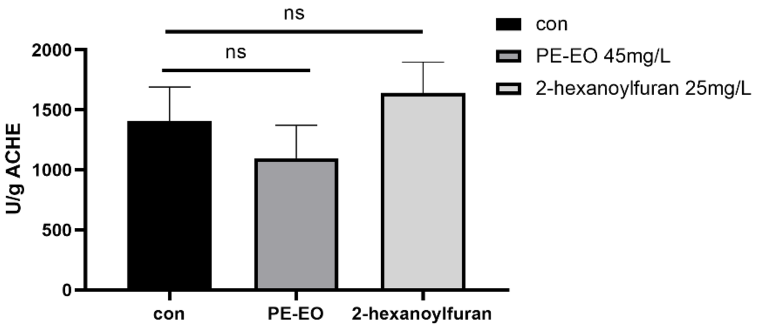

Figure S2. The acetylcholinesterase (AChE) activity of third instar larvae of *Cx. pipiens pallens* after treatment with Perilla essential oil (PE-EO) and 2-hexanoylfuran. The data are given as Mean±SD. Abbreviation: ns, not signifcant (P>0.05) compared with the control according to Student's t-test.
